# Wearable EEG monitoring reveals changes in rapid eye movement sleep in mild cognitive impairment

**DOI:** 10.1002/alz70857_100938

**Published:** 2025-12-24

**Authors:** Mason Taylor, Marc Goodfellow, Karin Petrini, Elizabeth Coulthard, George Stothart

**Affiliations:** ^1^ University of Bath, Bath, United Kingdom; ^2^ University of Exeter, Exeter, United Kingdom; ^3^ University of Bristol, Bristol, United Kingdom; ^4^ Bristol Medical School, University of Bristol, Bristol, United Kingdom; ^5^ North Bristol NHS Trust, Bristol, United Kingdom

## Abstract

**Background:**

Identifying reliable early biomarkers for Alzheimer's disease (AD) is critical for developing effective preventive strategies. Sleep disturbances, particularly in slow wave sleep (SWS) and rapid eye movement (REM) sleep, have been linked to AD pathology, including amyloid deposition and basal forebrain degeneration—changes that occur years before the onset of dementia. Several in‐lab polysomnography (PSG) studies indicate that deficits in SWS and REM sleep emerge early in the course of AD, notably in patients with mild cognitive impairment (MCI). However, limited accessibility, high costs, and poor patient tolerance significantly hinder the widespread adoption of PSG in both research and clinical settings. Wearable electroencephalography (EEG) offers a more feasible alternative for assessing sleep architecture in the home environment. Here, we used wearable EEG to assess changes in sleep stages in MCI and investigate their association with cognitive functioning.

**Method:**

A preliminary cohort of 7 patients with MCI due to probable AD and 13 age‐matched cognitively healthy controls underwent at‐home sleep monitoring for up to six consecutive nights using the Sleep Profiler headband, a single‐channel frontal EEG device. Global cognitive functioning was assessed using the Montreal Cognitive Assessment (MoCA). Sleep was scored using the U‐Sleep deep learning algorithm, validated against technician‐scored PSG data. Sleep stages were compared between groups and correlated with MoCA scores. Data collection is ongoing and further data will be presented at the conference.

**Result:**

Patients with MCI spent a significantly lower percentage of their total sleep time in REM sleep compared to controls (*p* = 0.043, *d* = 1.021), and reduced REM sleep percentage was significantly associated with lower MoCA scores (*p* = 0.040). No other sleep stages differed between the groups.

**Conclusion:**

Reduced REM sleep, which is detectable using wearable EEG, may serve as an early biomarker of AD and is associated with cognitive decline. These findings highlight the potential of non‐invasive, at‐home sleep monitoring for identifying those at the earliest symptomatic stage of AD.

